# Supplementary material for: Identification of photoperiod-induced specific miRNAs in the adrenal glands of Sunite sheep (Ovis aries)
Source: Front Vet Sci. 2022 Jul 22;9:888207. doi: 10.3389/fvets.2022.888207 (PMC9354845; doi:10.3389/fvets.2022.888207)
Supplement: Supplementary file 1 [file Data_Sheet_1.ZIP › Supplementary file/miRDeep2 file.zip!/miRDeep2 file/Know_Structure/SP42A2/oar-mir-191.pdf]

miRBase precursor : oar-mir-191
Total read count : 131215
oar-miR-191 read count : 130883
remaining reads : 332

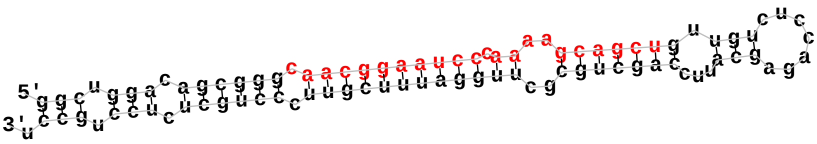

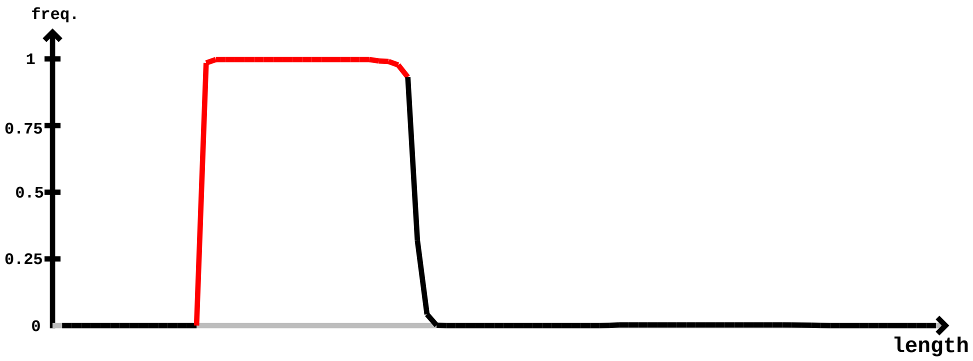

oar-miR-191

```
5'- ggcuggacagcgggcaacggaaucccaaaagcagcuguugucuccagagcauuccagcugcgcuuggauuucguucccugcucuccugccu   -3'   exp
    (((.(((.((((((.((((((((((.((..(((((((.(((.......)))...)))))))..))))))))))))).)))))).))).))).  reads    mm    sample
    ..............caacggaaucccaaaagc...........................................................  699      0     seq
    ..............caacggaaucccaaaagca..........................................................  246      0     seq
    ..............caacggaaucccaaaagcag.........................................................  1714     0     seq
    ..............caacggaaucccaaaagcagc........................................................  5915     0     seq
    ..............caacggaaucccaaaagcagcu.......................................................  79834    0     seq
    ..............caacggaaucccaaaagcagcug......................................................  35606    0     seq
    ..............caacggaaucccaaaagcagcugu.....................................................  5098     0     seq
    ..............caacggaaucccaaaagcagcuguu....................................................  111      0     seq
    ...............aacggaaucccaaaagc...........................................................  6        0     seq
    ...............aacUgaaucccaaaagc...........................................................  1        1     seq
    ...............aacggaaucccaaaagca..........................................................  1        0     seq
    ...............aacggaaucccaaaagcag.........................................................  7        0     seq
    ...............aacggaaucccaaaagcagc........................................................  40       0     seq
    ...............aacggaaucccaaaagcagcu.......................................................  558      0     seq
    ...............aacggaaucccaaaagcagcug......................................................  720      0     seq
    ...............aacggaaucccaaaagcagcugu.....................................................  310      0     seq
    ...............aacggaaucccaaaagcagcuguu....................................................  6        0     seq
    ................acggaaucccaaaagcagcu.......................................................  5        0     seq
    ................acggaaucccaaaagcagcug......................................................  5        0     seq
    ..................ggaaucccaaaagcagcu.......................................................  1        0     seq
    .....................................................ccagcugcgcuuggauuucguuc...............  1        0     seq
    .......................................................agcugcgcuuggauuucguuc...............  3        0     seq
    ........................................................gcugcgcuuggauuucguu................  11       0     seq
    ........................................................gcugcgcuuggauuucguuc...............  29       0     seq
    ........................................................gcugcgcuuggauuucguucc..............  21       0     seq
    ........................................................gcugcgcuuggauuucguuccc.............  11       0     seq
    ........................................................gcugcgcuuggauuucguucccu............  2        0     seq
    .........................................................cugcgcuuggauuucguu................  23       0     seq
    .........................................................cugcgcuuggauuucguuc...............  28       0     seq
    .........................................................cugcgcuuggauuucguucc..............  38       0     seq
    .........................................................cugcgcuuggauuucguuccc.............  106      0     seq
    .........................................................cugcgcuuggauuucguucccu............  51       0     seq
    ..........................................................ugcgcuuggauuucguuc...............  1        0     seq
    ..........................................................ugcgcuuggauuucguuccc.............  2        0     seq
    ..........................................................ugcgcuuggauuucguucccu............  5        0     seq
```

oar-miR-191

ggcuggacagcgggcaacggaaucccaaaagcagcuguugucuccagagcauuccagcugcgcuuggauuucguucccugcucuccugccu
